# Vibrotactile stimulation at gamma frequency mitigates pathology related to neurodegeneration and improves motor function

**DOI:** 10.3389/fnagi.2023.1129510

**Published:** 2023-05-18

**Authors:** Ho-Jun Suk, Nicole Buie, Guojie Xu, Arit Banerjee, Edward S. Boyden, Li-Huei Tsai

**Affiliations:** ^1^Picower Institute for Learning and Memory, Massachusetts Institute of Technology, Cambridge, MA, United States; ^2^Department of Brain and Cognitive Sciences, Massachusetts Institute of Technology, Cambridge, MA, United States; ^3^Media Arts and Sciences, Massachusetts Institute of Technology, Cambridge, MA, United States; ^4^McGovern Institute, Massachusetts Institute of Technology, Cambridge, MA, United States; ^5^Department of Biological Engineering, Massachusetts Institute of Technology, Cambridge, MA, United States; ^6^Koch Institute, Massachusetts Institute of Technology, Cambridge, MA, United States; ^7^Center for Neurobiological Engineering, Massachusetts Institute of Technology, Cambridge, MA, United States; ^8^Howard Hughes Medical Institute, Cambridge, MA, United States; ^9^Broad Institute of Harvard and Massachusetts Institute of Technology, Cambridge, MA, United States

**Keywords:** gamma frequency, tactile stimulation, vibration, neurodegeneration, motor function

## Abstract

The risk for neurodegenerative diseases increases with aging, with various pathological conditions and functional deficits accompanying these diseases. We have previously demonstrated that non-invasive visual stimulation using 40 Hz light flicker ameliorated pathology and modified cognitive function in mouse models of neurodegeneration, but whether 40 Hz stimulation using another sensory modality can impact neurodegeneration and motor function has not been studied. Here, we show that whole-body vibrotactile stimulation at 40 Hz leads to increased neural activity in the primary somatosensory cortex (SSp) and primary motor cortex (MOp). In two different mouse models of neurodegeneration, Tau P301S and CK-p25 mice, daily exposure to 40 Hz vibrotactile stimulation across multiple weeks also led to decreased brain pathology in SSp and MOp. Furthermore, both Tau P301S and CK-p25 mice showed improved motor performance after multiple weeks of daily 40 Hz vibrotactile stimulation. Vibrotactile stimulation at 40 Hz may thus be considered as a promising therapeutic strategy for neurodegenerative diseases with motor deficits.

## 1. Introduction

Aging is a major risk factor for neurodegeneration (Wyss-Coray, 2016; Hou et al., 2019), and aging-related brain pathologies such as DNA damage (Lu et al., 2004), synaptic loss (Morrison and Baxter, 2012), and accumulation of tau aggregates (Harrison et al., 2019) are often exacerbated in neurodegenerative diseases. For example, DNA breaks are more frequently observed in the post-mortem brains from patients with Alzheimer’s disease (AD), amyotrophic lateral sclerosis (ALS), or Parkinson’s disease (PD) compared to healthy controls (Madabhushi et al., 2014; Simpson et al., 2015; Shanbhag et al., 2019), and synapse loss is associated with sensory, motor, and cognitive impairments in a number of neurodegenerative disorders including AD and ALS (Kashyap et al., 2019; Subramanian and Tremblay, 2021; Holland et al., 2022). Increased tau aggregation is considered a hallmark pathology of several different neurodegenerative conditions—known as tauopathies—and can be triggered by mutations in the tau gene (MAPT; Wang and Mandelkow, 2016) or hyperphosphorylation of certain residues in the tau protein (Miao et al., 2019; Xia et al., 2020, 2021). Tauopathies include highly prevalent neurodegenerative diseases such as AD and frontotemporal dementia (FTD) as well as a number of other, rarer forms of neurodegeneration such as progressive supranuclear palsy (PSP) and Pick disease (Wang and Mandelkow, 2016). Recent studies have also implicated tau-related pathology in neurodegenerative diseases not traditionally known as tauopathies, such as PD; about 50% of PD patients show tau tangles (Zhang et al., 2018; Henderson et al., 2019), and single-nucleotide polymorphisms in the tau gene have been identified as risk factors for PD (Edwards et al., 2010). Clinical manifestations of tauopathies include motor dysfunction in addition to cognitive impairment, and while motor dysfunction is more prominently observed in diseases such as PD and PSP, it also occurs in other tauopathies such as FTD and AD that predominantly show cognitive deficits (Ganguly and Jog, 2020; Wiesman et al., 2021; Schönecker et al., 2022).

We have previously shown that non-invasive sensory stimulation using a light flickering or an auditory tone repeating at 40 Hz induces gamma oscillations (which we call Gamma ENtrainment Using Sensory stimulation or GENUS) and reduces pathological features in the brains of different mouse models of neurodegeneration. For example, we found that visual or auditory GENUS reduced phosphorylated tau in the respective sensory cortices of Tau P301S tauopathy model mice (Allen et al., 2002) when used for 1 h/day for 7 days (Iaccarino et al., 2016; Martorell et al., 2019), and that visual GENUS ameliorated neuronal loss when used for 3 weeks (Adaikkan et al., 2019). In CK-p25 mice, an inducible mouse model of neurodegeneration (Cruz et al., 2003), 6 weeks of 1 h/day visual GENUS reduced DNA double-strand breaks, synaptic loss, and neurodegeneration (Adaikkan et al., 2019). In both mouse models of neurodegeneration, daily visual GENUS over multiple weeks was also found to improve spatial memory (Adaikkan et al., 2019).

Other studies have reported that non-invasive sensory stimulation using another modality, namely somatosensory stimulation using vibrotactile stimuli, can lead to beneficial effects on motor performance. For example, whole-body vibration at gamma frequencies was found to improve motor function both in aged rodents (Boerema et al., 2018; Oroszi et al., 2022a,b) and in human patients with stroke (van Nes et al., 2006) or PD (Mosabbir et al., 2020). However, it remains unclear whether vibrotactile stimulation at gamma frequencies can also reduce pathological features in the brain, similar to visual and auditory GENUS.

Here, we investigated the effect of whole-body vibrotactile stimulation at 40 Hz on brain pathology and motor function in mouse models of neurodegeneration. We found that vibrotactile stimulation at 40 Hz induced neural activity both in the primary sensory cortex (SSp) and primary motor cortex (MOp). We also show that multi-week, daily vibrotactile stimulation ameliorated pathology in these brain regions, reducing phosphorylated tau and neurodegeneration in Tau P301S mice and decreasing synaptic protein loss and DNA damage in CK-p25 mice. In addition, we observed improved performance on motor tasks in both mouse models following multi-week, daily vibrotactile stimulation.

## 2. Materials and methods

### 2.1. Animals

All animal work was approved by the Committee for Animal Care of the Division of Comparative Medicine at the Massachusetts Institute of Technology. Male C57BL/6 J mice were obtained from the Jackson laboratory. All transgenic mice were bred and maintained in our animal facility. Tau P301S mice used in our experiments were 9-month-old male mice. CK-p25 mice used in our experiments were 6-month-old female mice. CK-p25 mice were initially raised on a doxycycline-containing diet to repress p25 expression and then switched to a normal rodent diet to induce p25 transgene expression over 6 weeks during the stimulation experiments. Two to five mice were housed in a single cage, on a standard 12-h light/12-h dark cycle. All experiments were performed during the light cycle. Food and water were provided without restriction.

### 2.2. Vibrotactile stimulation

**Figure 1:**
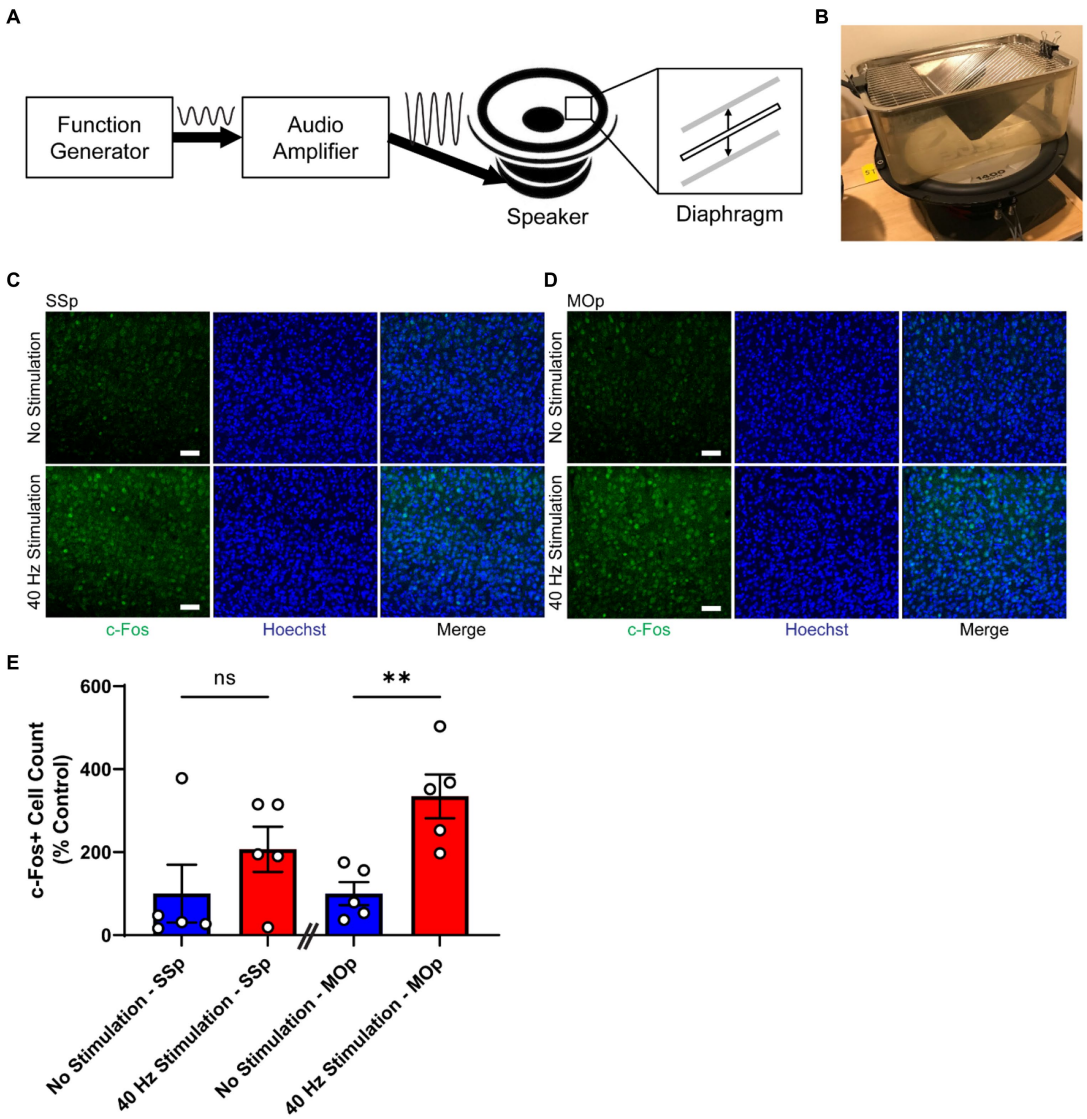
40 Hz vibrotactile stimulation induces neural activity in SSp and MOp. **(A)** Schematic representation of the vibrotactile stimulation system, composed of a function generator that produces an electrical signal at 40 Hz, an audio amplifier that amplifies the signal, and a speaker that converts the amplified signal to a physical movement of a diaphragm. The diaphragm moves up and down to create vibration at 40 Hz. Sine wave to the right of the “Function Generator” box represents the electrical signal at 40 Hz generated by the function generator, and the sine wave to the right of the “Audio Amplifier” box represents the amplified version of the 40 Hz signal. Thick solid arrows indicate the direction of signal flow. Thin vertical arrows represent the direction of the diaphragm movement. **(B)** Photograph of a mouse cage placed on top of a speaker that is generating 40 Hz vibration. **(C,D)** Representative immunohistochemistry images showing c-Fos antibody (green) and Hoechst labeling of cell nucleus (blue) in SSp **(C)** or MOp **(D)** of 4-month-old C57BL/6 J mice after 1 h of no stimulation or 40 Hz vibrotactile stimulation. Scale bar, 50 μm. **(E)** Number of c-Fos positive cells in SSp and MOp of 4-month-old C57BL/6 J mice after 1 h of no stimulation or 40 Hz vibrotactile stimulation, normalized to the average of no stimulation controls (*n* = 5 mice per group). ^**^p < 0.01, ns, not significant; unpaired *t*-test. Each circle represents an individual mouse, each bar represents the mean, and error bars represent SEM.

40 Hz vibrotactile stimulation was delivered using an acoustic system composed of a function generator, an audio amplifier (BOSS Audio Systems, PF2600), and a full-range speaker (BOSS Audio Systems, CX122) that converts a 40 Hz electrical sinusoidal signal to a corresponding 40 Hz vertical vibration of a diaphragm (Figure 1A). Teensy 3.6 Development Board (PJRC) was used as the function generator, programmed to output the 40 Hz sinusoidal signal through its analog output channel.

The stimulation room was set up with multiple speakers, with half of the speakers connected to the audio amplifier and another half not connected to the audio amplifier. These two groups of speakers were placed in an alternating pattern (Supplementary Figure 1) so that mice in the stimulation group and no stimulation group could all experience a similar amount of low humming noise (close to E_1_) generated by the vibrating speakers.

An empty mouse cage with a metal wire cage lid (identical to the home cage but without bedding, food, or water) was placed on top of each speaker near its center (Figure 1B). To prevent the cage from falling off the vibrating speaker during vibration, the cage and lid were held in place by two rubber bands that were clamped to the top of the lid on one end and the rim of the speaker on the other end (Figure 1B). The vibration inside the vibrating cage was at 40 Hz with about 16 μm displacement, as measured by an accelerometer.

On each day of the stimulation, mice were moved from their holding room to the stimulation room, and they were habituated for 30 min in the stimulation room under dim light. After habituation, mice were introduced to the cages on top of the speakers (Figure 1B), one mouse per cage. A mouse in the stimulation group was placed inside the cage on top of a speaker connected to the audio amplifier, and a mouse in the no stimulation group was placed inside the cage on top of a speaker not connected to the audio amplifier. After 1 h of stimulation or no stimulation, mice were returned to their home cage and moved back to their holding room.

All of the mice that were studied for c-Fos underwent the hour-long stimulation or no stimulation session at around the same time of day (between noon and 2 PM). Animal handling was minimized before and after the session to reduce any impact on c-Fos expression.

### 2.3. Rotarod test

Mice were tested using a rotarod device (Med Associates Inc., ENV-574 M) that had a knurled rod (3.2 cm in diameter) divided into five 8 cm-wide lanes, which allowed up to five mice to be tested simultaneously. Each mouse completed three trials, and for each trial, individual mice were placed on the rod within one of the lanes, and the rod was accelerated from 4 to 40 rotations per minute over 5 min. The latency to fall was captured by the infrared fall detection sensor located just above the holding chamber (located 30 cm below the rod, for each lane). If a mouse clung to the rod instead of falling, the time at which the mouse completed the first full passive rotation was recorded as the latency to fall. After completing each trial, mice were kept in the holding chambers for at least 1 min before starting the next trial. The rod and holding chambers were cleaned with 70% ethanol after all three trials were completed and air-dried before starting a new trial for the next group of mice.

### 2.4. Grid hang test

Each mouse was placed on a wire grid (36 × 14.5 cm), and the grid was slowly inverted. The inverted grid was placed about 50 cm above a cage filled with bedding to provide cushioning when a mouse fell. The time between the grid inversion and mouse falling from the grid was recorded as the latency to fall.

### 2.5. Immunohistochemistry

For immunohistochemistry (IHC) with c-Fos antibody, perfusion was started about 1 h after the stimulation or no stimulation session. For all other IHC, perfusion was started about 24 h after the last daily stimulation or no stimulation session. Mice were anesthetized and transcardially perfused with 40 mL of ice-cold PBS. Brains were collected and post-fixed for 24 h in 4% paraformaldehyde at 4°C. They were then transferred to PBS and stored at 4°C until sectioning. Brains were sectioned by mounting them in 3% agarose in PBS and slicing into 40 μm sections using a vibratome (Leica VT1000S). For immunostaining, brain slices were permeabilized and blocked by incubating them in a blocking buffer (5% normal donkey serum, 0.3% Triton-X in PBS) for 2 h at room temperature on a shaker. Blocking buffer was removed, and slices were incubated in a fresh blocking buffer containing primary antibody for two nights at 4°C on a shaker. The primary antibodies included anti-c-Fos (1:400 dilution; Millipore Sigma, ABE457), anti-phosphorylated tau S396 (1:2,000 dilution; Cell Signaling Technologies, 9632), anti-NeuN (1:500 dilution; Synaptic Systems, 266004), anti-GABBR1 (1:500 dilution; Thermo Fisher Scientific, MA5-27704), anti-vGlut1 (1:500 dilution, Synaptic Systems, 135302), and anti-γH2Ax (1:500 dilution; Millipore Sigma, 05636). Following the incubation with primary antibody, slices were washed three times with PBS (10 min for each wash) at room temperature and then incubated in a fresh blocking buffer containing secondary antibodies conjugated with Alexa Fluor 488, 555, 594, or 647 (1:500 dilution; Invitrogen) and Hoechst 33342 nuclear stain (1:10,000 dilution; Thermo Fisher Scientific, H3570) for 2 h at room temperature on a shaker. Slices were then washed three times with PBS (10 min for each wash) at room temperature and mounted to slides using Fluoromount-G (Electron Microscopic Sciences).

### 2.6. Imaging and quantification

Images were acquired using LSM 710 or LSM 880 confocal microscope (Zeiss) with a 20x or 40x objective. The region of interest (ROI) spanned across layer 1 and the upper part of layer 2/3 for phosphorylated tau S396 imaging, and across layer 2/3 for the rest of the imaging (Supplementary Figure 2). Two sections were used per mouse for each experiment, and each of the entire 40 μm sections was imaged in a *z* stack with a 1 μm step size. Analysis was performed using Imarisx64 9.8.1 (Bitplane, Zurich, Switzerland) and ImageJ 1.53q. C-Fos and NeuN-positive cell counts were performed automatically using the Imaris spot function, only counting cells that were also positive for Hoechst. Consistent parameters were used, thresholding for signal quality and intensity. Phosphorylated tau S396 and γH2Ax area were measured using the Imaris surface function. Consistent parameters were used, thresholding by signal intensity. Phosphorylated tau S396 and γH2Ax-positive cell counts were performed manually with Image J using the cell counter, only counting cells that were also positive for NeuN (Supplementary Figure 3). GABBR1, vGlut1, and γH2Ax signal intensity were measured using Image J.

### 2.7. Statistical analysis

Statistical analysis was performed using Prism 9 software (GraphPad). Statistical significance between stimulated and non-stimulated groups for immunohistochemistry results and the grid hang test was calculated using a two-tailed unpaired *t*-test. Statistical significance between stimulated and non-stimulated groups for the rotarod test was calculated using two-way ANOVA with Šídák’s multiple comparisons test.

## 3. Results

### 3.1. 40 Hz vibrotactile stimulation induces neural activity in SSp and MOp

Previous studies have shown that neural responses can be induced in the somatosensory domain of the brain by applying periodic vibrotactile stimulation to a specific location of the body, such as fingertips in humans (Langdon et al., 2011; Ross et al., 2013; Jamali and Ross, 2014) or whiskers in rodents (Garabedian et al., 2003; Khatri et al., 2004; Ewert et al., 2008). To determine whether tactile stimulation at 40 Hz delivered as whole-body vibration can induce neural activity in the SSp and MOp, we subjected 4-month-old C57BL/6 J mice to either 40 Hz vibration or no vibration for 1 h using our vibrotactile stimulation system (Figures 1A,B) and performed immunostaining for c-Fos (Figures 1C,D), a widely used molecular marker for neural activity (Chung, 2015). We found that mice exposed to 40 Hz vibrotactile stimulation showed a trend toward a higher number of c-Fos-positive cells in the SSp (with an increase of about 2.1-fold) and significantly more c-Fos-positive cells in the MOp (with an increase of about 3.3-fold) compared to no stimulation controls (Figure 1E). These findings suggest that 40 Hz tactile stimulation using whole-body vibration can induce neural activity in both the SSp and MOp.

### 3.2. 40 Hz vibrotactile stimulation reduces multiple pathologies in SSp and MOp of Tau P301S and CK-p25 mice

To study the effect of our 40 Hz vibrotactile stimulation on neurodegenerative disease-related pathologies in the SSp and MOp, we used two different mouse models of neurodegeneration, Tau P301S and CK-p25 mice. Tau P301S mice harbor a transgene expressing human tau with the FTD-associated P301S mutation (Allen et al., 2002) and display widespread tau pathology as well as neurodegeneration (López-González et al., 2015; Koivisto et al., 2019). CK-p25 mice develop severe losses in neurons (Cruz et al., 2003) and synaptic proteins (Fischer et al., 2005) as well as an increase in DNA damage (Kim et al., 2008; Adaikkan et al., 2019) after long-term (typically 6 weeks of) p25 induction.

**Figure 2:**
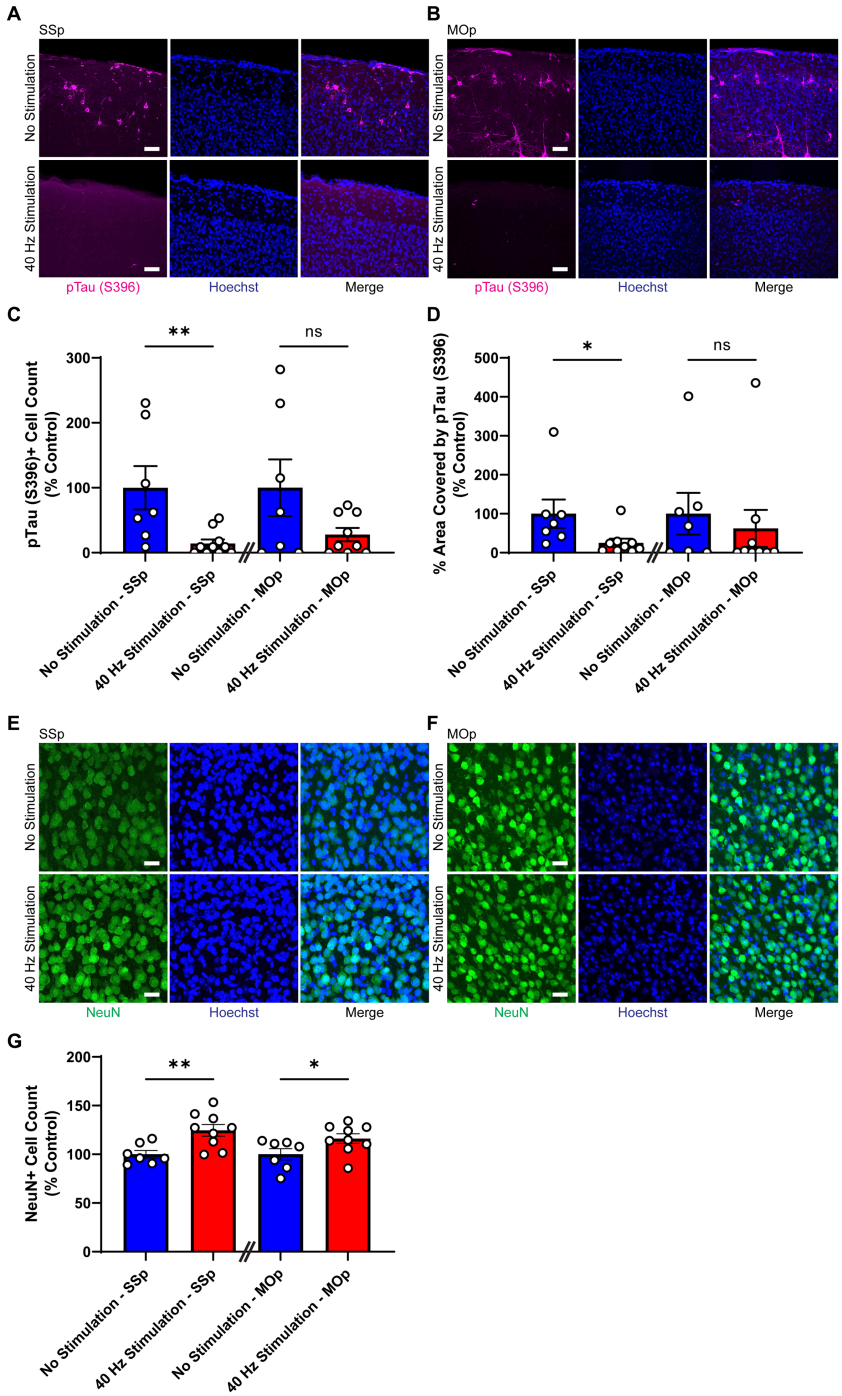
40 Hz vibrotactile stimulation reduces phosphorylated tau and neuronal loss in SSp and MOp of Tau P301S mice. **(A,B)** Representative immunohistochemistry images showing anti-pTau (S396) antibody (magenta) and Hoechst labeling of cell nucleus (blue) in SSp **(A)** or MOp **(B)** of 9-month-old Tau P301S mice after 21 days of no stimulation or 40 Hz vibrotactile stimulation, 1 h/day. Scale bar, 50 μm. **(C)** Number of pTau (S396)-positive cells in SSp and MOp of 9-month-old P301S mice after 21 days of no stimulation or 40 Hz vibrotactile stimulation, 1 h/day, normalized to the average of no stimulation controls (*n* = 7 mice in No Stimulation—SSp, 10 mice in 40 Hz Stimulation—SSp; *n* = 7 mice in No Stimulation—MOp, and 9 mice in 40 Hz Stimulation—MOp). ^**^p < 0.01, ns, not significant; unpaired *t*-test. **(D)** Same as **(C)** for percentage of area covered by pTau (S396), normalized to the average of no stimulation controls (*n* = 7 mice in No Stimulation—SSp, 9 mice in 40 Hz Stimulation—SSp; *n* = 7 mice in No Stimulation—MOp, 9 mice in 40 Hz Stimulation—MOp). ^*^p < 0.05, ns, not significant; unpaired *t*-test. **(E,F)** Representative immunohistochemistry images showing NeuN antibody (green) and Hoechst labeling of cell nucleus (blue) in SSp **(E)** or MOp **(F)** of 9-month-old P301S after 21 days of no stimulation or 40 Hz vibrotactile stimulation, 1 h/day. Scale bar, 25 μm. **(G)** Number of NeuN-positive cells in SSp and MOp of 9-month-old P301S mice after 21 days of no stimulation or 40 Hz vibrotactile stimulation, 1 h/day, normalized to the average of no stimulation controls (*n* = 7 mice in No Stimulation—SSp, 9 mice in 40 Hz Stimulation—SSp; 7 mice in No Stimulation—MOp, and 9 mice in 40 Hz Stimulation—MOp). ^*^p < 0.05, ^**^p < 0.01; unpaired *t*-test. Each circle represents an individual mouse, each bar represents the mean, and error bars represent SEM.

First, we subjected 9-month old Tau P301S mice to 21 days of 40 Hz vibrotactile stimulation or no stimulation for 1 h/day. The duration of stimulation was based on results from an earlier study using 40 Hz visual stimulation on the same mouse model (Adaikkan et al., 2019). At this age, these mice exhibit pronounced accumulation of hyperphosphorylated tau in the somatosensory cortex and multiple other brain regions (López-González et al., 2015). Using immunostaining, we found that mice exposed to 40 Hz vibrotactile stimulation showed a reduction in phosphorylated tau S396, pTau (S396), compared to no stimulation controls (Figures 2A,B). Specifically, the number of pTau (S396)-positive cells was significantly reduced in the SSp (by 85.8%) and trended downward in the MOp (by 72.1%; Figure 2C). Similarly, the area covered by pTau (S396) was significantly reduced in the SSp (by 74.8%) and trended downward in the MOp (by 37.7%; Figure 2D). Neurodegeneration in the SSp and MOp was also reduced, as assessed by immunostaining for the neuronal marker NeuN (Figures 2E,F), with a significantly higher number of NeuN-positive cells observed both in the SSp (124.6%) and MOp (116.1%) of stimulated mice compared to no stimulation controls (Figure 2G). These results show that vibrotactile stimulation at 40 Hz can ameliorate accumulation of phosphorylated tau and neuronal loss in the SSp and MOp of the Tau P301S mouse model of tauopathy.

**Figure 3:**
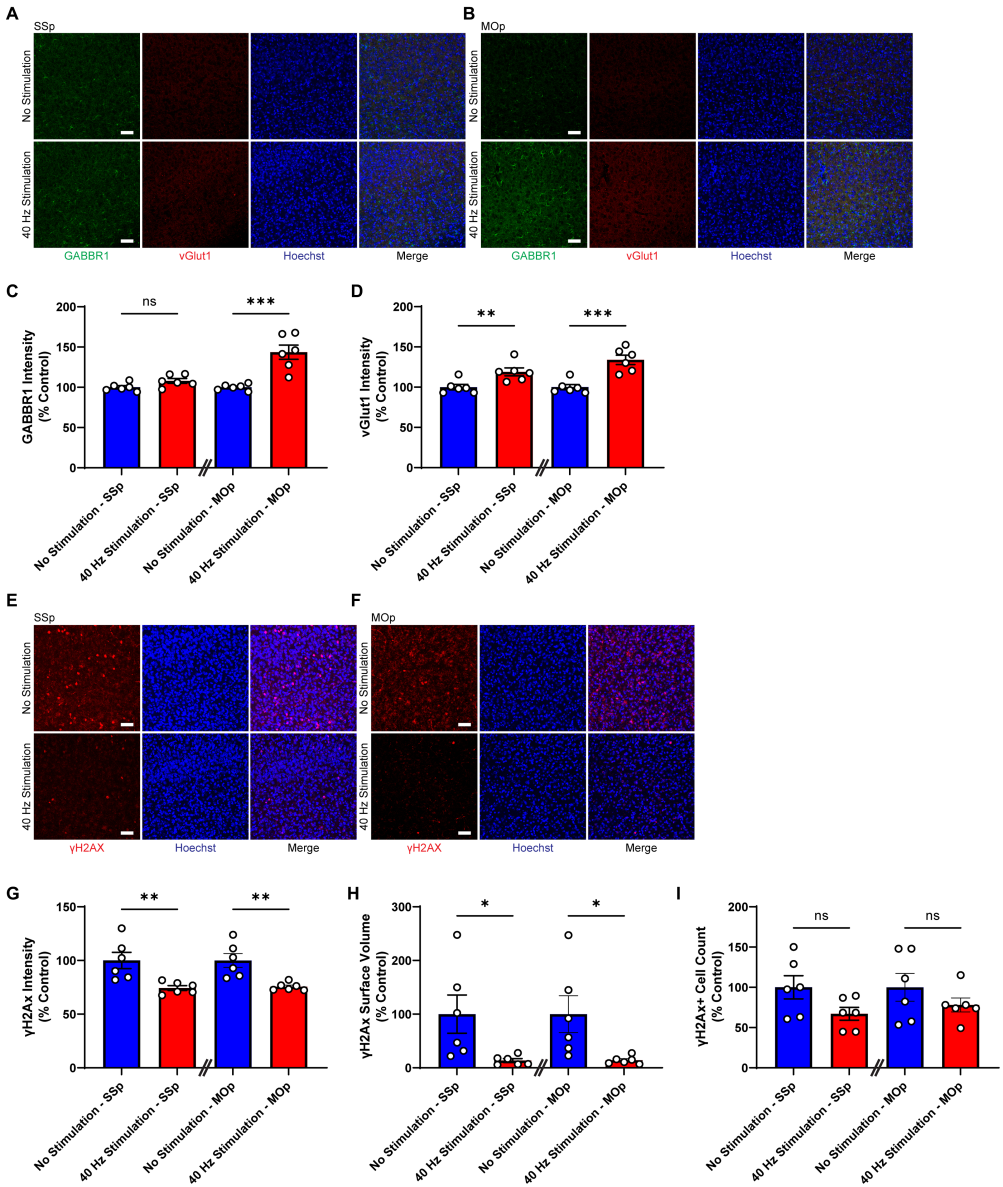
40 Hz vibrotactile stimulation preserves synaptic proteins and reduces DNA damage in SSp and MOp of CK-p25 mice. **(A,B)** Representative immunohistochemistry images showing GABBR1 antibody (green), vGlut1 antibody (red), and Hoechst labeling of cell nucleus (blue) in SSp **(A)** or MOp **(B)** of 6-month-old CK-p25 mice after 42 days of no stimulation or 40 Hz vibrotactile stimulation, 1 h/day. Scale bar, 50 μm. **(C)** GABBR1 mean intensity in SSp and MOp of 6-month-old CK-p25 mice after 42 days of no stimulation or 40 Hz vibrotactile stimulation, 1 h/day, normalized to the average of no stimulation controls (*n* = 6 mice per group). ^***^p < 0.001, ns, not significant; unpaired *t*-test. **(D)** Same as **(C)** for vGlut1 mean intensity, normalized to the average of no stimulation controls (*n* = 6 mice per group). ^**^p < 0.01, ^***^p < 0.001; unpaired *t*-test. **(E,F)** Representative immunohistochemistry images showing anti-γH2Ax antibody (red) and Hoechst labeling of cell nucleus (blue) in SSp **(E)** or MOp **(F)** of 6-month-old CK-p25 mice after 42 days of no stimulation or 40 Hz vibrotactile stimulation, 1 h/day. Scale bar, 50 μm. **(G)** γH2Ax signal intensity in SSp and MOp of 6-month-old CK-p25 mice after 42 days of no stimulation or 40 Hz vibrotactile stimulation, 1 h/day, normalized to the average of no stimulation controls (*n* = 6 mice per group). ^**^p < 0.01; unpaired *t*-test. **(H)** Same as **(G)** for the total surface volume of γH2Ax signal, normalized to the average of no stimulation controls (*n* = 6 mice per group). ^*^p < 0.05; unpaired *t*-test. **(I)** Same as **(G)** for the number of γH2Ax-positive cells, normalized to the average of no stimulation controls (*n* = 6 mice per group). ns, not significant; unpaired *t*-test. Each circle represents an individual mouse, each bar represents the mean, and error bars represent SEM.

Next, we exposed 6-month-old CK-p25 mice to 6 weeks of 40 Hz vibrotactile stimulation or no stimulation for 1 h/day while simultaneously inducing p25 expression. A longer treatment period was used for CK-p25 mice compared to Tau P301S mice given the 6-week induction period for target brain pathologies in the CK-p25 mouse model, as it was done previously for 40 Hz visual stimulation (Adaikkan et al., 2019). Although mice exposed to stimulation did not show a significant difference in the number of NeuN-positive cells in the SSp and MOp compared to no stimulation controls (Supplementary Figure 4), immunostaining for synaptic proteins gamma-aminobutyric acid B receptor subunit 1 (GABBR1) and vesicular glutamate transporter 1 (vGlut1; Figures 3A,B) revealed a significantly higher signal intensity for these transporters in stimulated mice compared to no stimulation controls (Figures 3C,D); in the SSp, GABBR1 showed a trend toward an increase in signal intensity (by 107.9%) and vGlut1 showed a significant increase in signal intensity (by 119.0%); in the MOp, both GABBR1 and vGlut1 showed a significant increase in signal intensity (by 143.6% for GABBR1; by 133.9% for vGlut1). We also saw a significant reduction in γH2Ax, a marker for DNA double-strand breaks (Xiao et al., 2013), in the SSp and MOp of stimulated mice compared to no stimulation controls (Figures 3E,F), both in terms of signal intensity (reduction by 25.6% in SSp and 24.5% in MOp; Figure 3G) and surface volume positive for γH2Ax (reduction by 86.5% in SSp and 85.9% in MOp; Figure 3H). In addition, the number of γH2Ax-positive cells was non-significantly reduced in the SSp (by 32.9%) and MOp (by 21.9%) of stimulated mice (Figure 3I). Together, these results suggest that vibrotactile stimulation at 40 Hz can preserve synaptic proteins and reduce DNA damage in the SSp and MOp of the CK-p25 mouse model of neurodegeneration.

### 3.3. 40 Hz vibrotactile stimulation improves motor performance in Tau P301S and CK-p25 mice

We also investigated the effect of our 40 Hz vibrotactile stimulation on motor function using Tau P301S mice, which are known to develop motor impairment of the hind limbs and generalized muscle weakness (Yoshiyama et al., 2007; López-González et al., 2015; Koivisto et al., 2019), and CK-p25 mice, for which motor dysfunction has not been reported. We subjected both mouse models to two tests of motor function, the rotarod test and the grid-hang test. The rotarod test is a widely used tool for assessing motor coordination and balance that is implemented by measuring the time that an animal is able to stay on a rotating rod over multiple trials (Brooks et al., 2012). The grid hang test determines static muscle strength by placing a mouse on a wire grid, inverting the grid, and measuring the latency to fall (Koivisto et al., 2019).

**Figure 4:**
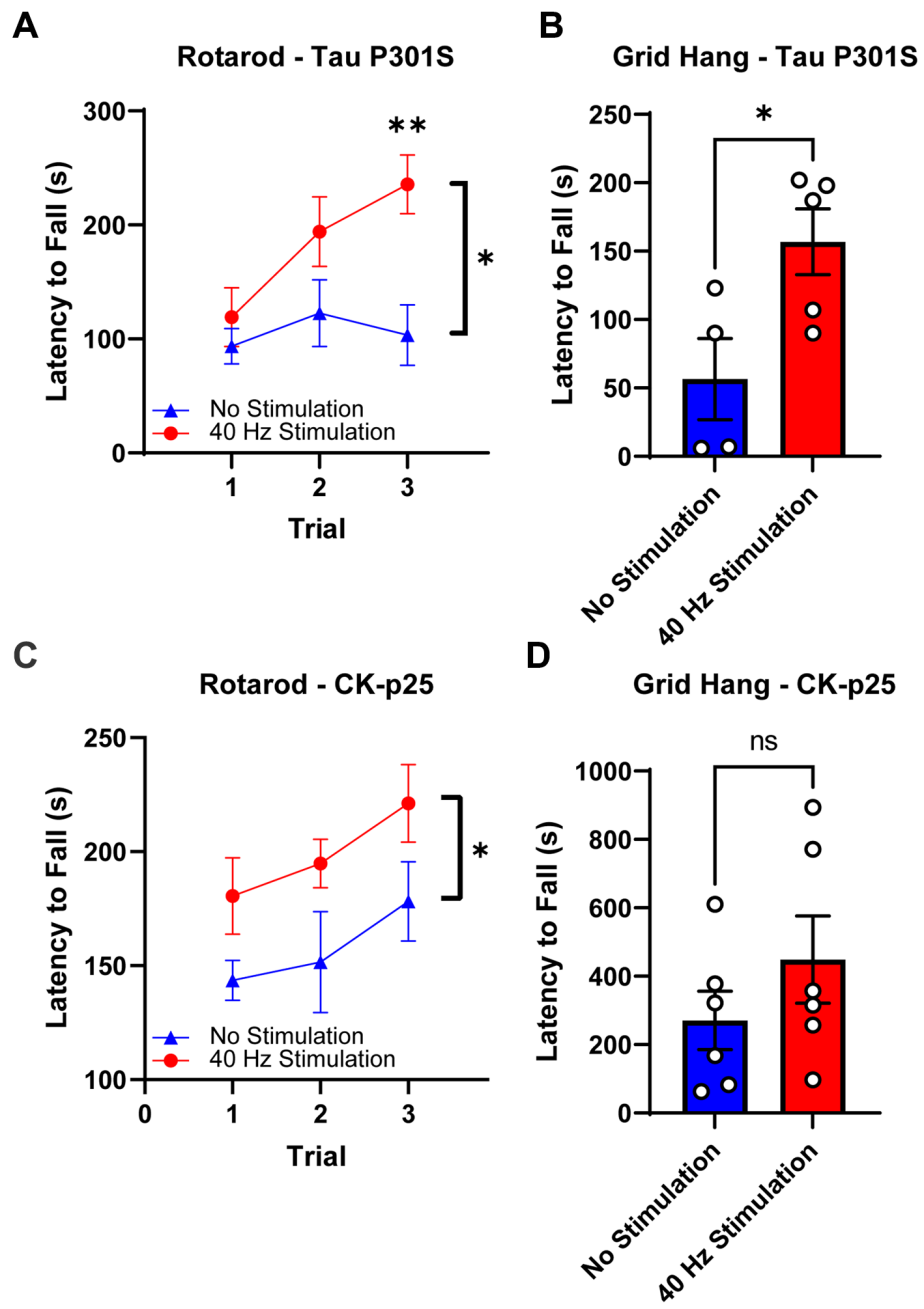
40 Hz vibrotactile stimulation improves performance on motor tasks in Tau P301S and CK-p25 mice. **(A)** Latency to fall during the rotarod test over three trials for 9-month-old P301S mice after 17 days of no stimulation or 40 Hz vibrotactile stimulation, 1 h/day [*n* = 9 mice in No Stimulation (triangle), 10 mice in 40 Hz Stimulation (circle)]. ^*^p < 0.05 between groups across all three trials, ^**^p < 0.01 between groups at trial 3; two-way ANOVA with Šídák’s multiple comparisons test. Each filled shape represents the mean and error bars represent SEM for each group and each trial. **(B)** Latency to fall during grid hang test for 9-month-old P301S mice after 21 days of no stimulation or 40 Hz vibrotactile stimulation, 1 h/day (*n* = 4 mice in No Stimulation, 5 mice in 40 Hz Stimulation). ^*^p < 0.05; unpaired *t*-test. Each circle represents an individual mouse, each bar represents the mean, and error bars represent SEM. **(C)** Same as **(A)** for 6-month-old CK-p25 mice after 38 days of no stimulation or 40 Hz vibrotactile stimulation, 1 h/day [*n* = 6 mice in No Stimulation (triangle), 5 mice in 40 Hz Stimulation (circle)]. ^*^p < 0.05 between groups across all three trials; two-way ANOVA with Šídák’s multiple comparisons test. Each filled shape represents the mean and error bars represent SEM for each group and each trial. **(D)** Same as **(B)** for 6-month-old CKp25 mice after 39 days of no stimulation or 40 Hz vibrotactile stimulation, 1 h/day (*n* = 6 mice in No Stimulation, 6 mice in 40 Hz Stimulation). ns, not significant; unpaired t-test. Each circle represents an individual mouse, each bar represents the mean, and error bars represent SEM.

After 17 days of 40 Hz vibrotactile stimulation or no stimulation, stimulated 9-month-old Tau P301S mice showed a significant increase (171.7%) in their latency to fall from the rotating rod over three trials compared to no stimulation controls (Figure 4A). After 21 days of 40 Hz vibrotactile stimulation or no stimulation, stimulated Tau P301S mice also performed better at the grid hang test, hanging onto the inverted grid significantly longer (277.5%) compared to no stimulation controls (Figure 4B). 40 Hz vibrotactile stimulation also led to improvements in motor function in 6-month-old CK-p25 mice, although to a lesser degree than in Tau P301S mice. CK-p25 mice showed a significant increase in their latency to fall during the rotarod test across three trials (126.1%) following 38 days of stimulation compared to no stimulation controls (Figure 4C), and they also exhibited a non-significant increase in their latency to fall from the inverted grid (165.7%) after 39 days of stimulation compared to no stimulation controls (Figure 4D). These data suggest that 40 Hz vibrotactile stimulation can improve performance on motor tasks in mouse models of neurodegeneration.

## 4. Discussion

In our study, we showed that 40 Hz tactile stimulation using whole-body vibration effectively induced neural activity in the SSp as well as the MOp and that long-term exposure to the daily stimulation had a marked impact on pathological features in these brain regions in mouse models of neurodegeneration. Specifically, we demonstrated that daily treatment with 40 Hz vibrotactile stimulation over the course of multiple weeks led to a decrease in phosphorylated tau accumulation and neuronal loss in the SSp and MOp of Tau P301S mice and a reduction in DNA double-strand breaks and loss of synaptic proteins in the same brain regions of CK-p25 mice. In both mouse models, 40 Hz vibrotactile stimulation also led to improvements in motor function.

Locally applied vibrotactile stimulation has been shown to induce phase-locked neural responses. For example, magnetoencephalogram (MEG) recordings have been used to demonstrate that stimulating the fingertips of human subjects with a plastic membrane fluttering at 20 Hz can lead to 20 Hz neural oscillations in the somatosensory area (Ross et al., 2013; Jamali and Ross, 2014). In anesthetized rats, a periodic whisker deflection can result in a phase-locked spiking response from neurons in the barrel cortex for stimulation frequencies in the gamma range, including 40 Hz (Garabedian et al., 2003; Khatri et al., 2004). For whole-body vibration, phase-locked neural responses have not been directly shown, likely due to the technical difficulty in obtaining stable, low-noise electrophysiological recordings while vibrating the whole body. In a previous study using 40 Hz visual stimulation, we observed robust gamma entrainment in brain regions that showed an increase in c-Fos-positive cells (Adaikkan et al., 2019), which suggests that induced neural activity captured by the c-Fos signal likely represented gamma oscillations in these brain regions. The increase in c-Fos-positive cells in the SSp and MOp of mice exposed to our 40 Hz vibrotactile stimulation may thus suggest that the stimulation could have induced gamma oscillations in these brain regions as well.

We found that the impact of our vibrotactile stimulation was not limited to the SSp, but also extended to the MOp. In addition to an increase in c-Fos-positive cells, both the SSp and MOp showed reductions in hyperphosphorylated tau, neuronal loss, DNA damage, and loss of synaptic proteins following 40 Hz vibrotactile stimulation. Others have also reported the effect of whole-body vibration at beta and gamma frequencies spreading to brain regions beyond the somatosensory cortex. In a clinical study with healthy adult participants, functional near-infrared spectroscopy was used to show that oxygenated hemoglobin concentration was increased in multiple cortices (e.g., primary motor cortex, premotor cortex, and supplementary motor cortex) in addition to the somatosensory cortex during 27 Hz stimulation using a whole-body vibration exercise platform (Choi et al., 2019), suggesting that the vibrotactile stimulation led to a widespread cortical activation. In another study involving elderly subjects with mild dementia, 8 weeks of whole-body vibration (starting at 20 Hz and increasing by 5 Hz every 2 weeks) resulted in an increase in electroencephalogram (EEG) activation across frontal, temporal, and parietal electrodes (Kim and Lee, 2018). These findings, combined with our current study as well as previous studies using visual or auditory GENUS (Iaccarino et al., 2016; Adaikkan et al., 2019; Martorell et al., 2019), indicate that the effect of non-invasive sensory stimulation can extend beyond the primary sensory cortex. While we do not currently know how widely the beneficial effects of 40 Hz vibrotactile stimulation can spread in the brain, visual, auditory, and combined visual and auditory GENUS have been shown to reduce brain pathology across different cortical areas including the prefrontal cortex and deeper regions like the hippocampus (Adaikkan et al., 2019; Martorell et al., 2019), suggesting that such spread in neuroprotective effects may also be feasible with vibrotactile stimulation alone or when combined with other sensory modalities. Of note, for the current study, we focused our analyses on the superficial layers of SSp and MOp (i.e., layers 1 and 2/3; Supplementary Figure 2), and thus cannot exclude the possibility of layer-specific differences in the degree of neuroprotection provided by 40 Hz vibrotactile stimulation.

Given the importance of the somatosensory cortex for motor function (Borich et al., 2015; Ingemanson et al., 2019; Noohi et al., 2019), we also studied the effect of our 40 Hz vibrotactile stimulation on motor coordination and balance (using the rotarod test) as well as muscle strength (using the grid hang test) after multiple weeks of daily stimulation. In Tau P301S mice, which have been shown to display motor deficits and muscle weakness (Yoshiyama et al., 2007; López-González et al., 2015; Koivisto et al., 2019), we observed significant improvements in both rotarod and grid-hang test performance. CK-p25 mice, which are not known to develop motor dysfunction, also showed improvements in both tests, but to a lesser degree than Tau P301S mice. Our findings are in line with results from previous rodent studies that demonstrated a beneficial effect of 30 Hz whole-body vibration on motor coordination, balance, and muscle strength in aged rats (Oroszi et al., 2022a,b), which show a decline in motor function (Altun et al., 2007; Ballak et al., 2014), and in young mice (Mettlach et al., 2014; Keijser et al., 2017). The underlying mechanisms of how our 40 Hz vibrotactile stimulation improves motor function are yet to be elucidated, but the reduction of pathological features in the somatosensory and motor cortices resulting from our stimulation is likely to be a contributing factor. In particular, synaptic protection, which was observed in CK-p25 mice (Figures 3A–D) and is likely to be present in Tau P301S mice based on the observed reduction of synaptotoxic tau pathology (Figures 2A–D), may have played a role in the preservation of motor function. Since we previously showed that pathology in a mouse model of AD was reduced by visual or auditory stimulation at 40 Hz, but not at other frequencies such as 8, 20, and 80 Hz or at random frequencies (Iaccarino et al., 2016; Martorell et al., 2019), the current study focused on vibrotactile stimulation at 40 Hz; however, the impact of vibrotactile stimulation at other frequencies on pathology and behavior remains to be elucidated. It is also worth noting the possibility that the 40 Hz humming noise generated by the vibrotactile device in the current study led to some level of neural entrainment and may have had an impact on brain pathology and motor function. However, since control animals were exposed to a similar amount of humming noise as animals undergoing vibrotactile stimulation, it is likely that 40 Hz vibrotactile stimulation accounted for most of the reduction in brain pathology and improvement in motor function observed in our current study.

The current study, along with our previous studies using visual or auditory GENUS (Iaccarino et al., 2016; Adaikkan et al., 2019; Martorell et al., 2019), demonstrates the possibility of using non-invasive sensory stimulation as a novel therapeutic strategy for ameliorating pathology and improving behavioral performance in neurodegenerative diseases. We recently completed a small-scale pilot study in which we found that long-term, at-home use of combined visual and auditory GENUS had no adverse effect in patients with mild AD while offering some beneficial effects such as reduction in brain atrophy and improvement in daily activity rhythmicity (Chan et al., 2022). More studies are required to determine whether (and to what extent) the beneficial effects of 40 Hz sensory stimulation observed in mouse models can be translated to human patients with neurodegenerative diseases, and if the type and degree of beneficial effects depend on the stimulation modality.

## Data availability statement

The raw data supporting the conclusions of this article will be made available by the authors, without undue reservation.

## Ethics statement

The animal study was reviewed and approved by Committee for Animal Care of the Division of Comparative Medicine at the Massachusetts Institute of Technology.

## Author contributions

H-JS, EB, and L-HT conceptualized and designed the project. H-JS, NB, GX, and AB performed stimulation experiments. H-JS, GX, and AB performed behavioral experiments. H-JS and NB performed immunostaining, imaging, image analysis, and statistical analysis. H-JS, NB, and L-HT wrote the manuscript. All authors contributed to the article and approved the submitted version.

## Funding

We would like to acknowledge the following individuals and foundations for their kind support of our work: The JPB Foundation, Halis Family Foundation, Eduardo Eurnekian, DeGroof-VM Foundation, The Dolby Family, Melissa Ko Hahn and Suguwu Douglas Hahn, Lester A. Gimpelson, Kathleen and Miguel Octavio, Glenda G. and Donald A. Mattes, Carol and Gene Ludwig Family Foundation, Eleanor Schwartz Charitable Foundation, David B. Emmes, Jay L. and Carroll Miller, Alan and Susan Patricof, Alan Alda, Willian C.L. and Bernadette Shih, Eduardo Bross, Anne Gao and Alex Hu, and Prisca Chen Marvin and Kim A. Marvin. EB would like to acknowledge Charles Hieken for kind support.

## Conflict of interest

L-HT and EB are co-Scientific Founders and serve on the scientific advisory board of Cognito Therapeutics, Inc.

The remaining authors declare that the research was conducted in the absence of any commercial or financial relationships that could be construed as a potential conflict of interest.

## Publisher’s note

All claims expressed in this article are solely those of the authors and do not necessarily represent those of their affiliated organizations, or those of the publisher, the editors and the reviewers. Any product that may be evaluated in this article, or claim that may be made by its manufacturer, is not guaranteed or endorsed by the publisher.
