# Supplementary material for: Vibrotactile stimulation at gamma frequency mitigates pathology related to neurodegeneration and improves motor function
Source: Front Aging Neurosci. 2023 May 18;15:1129510. doi: 10.3389/fnagi.2023.1129510 (PMC10233036; doi:10.3389/fnagi.2023.1129510)
Supplement: Supplementary file 1 [file Data_Sheet_1.docx]

Supplementary Material

Vibrotactile stimulation at gamma frequency mitigates pathology related to neurodegeneration and improves motor function

Ho-Jun Suk^1,2^^†^, Nicole Buie^1,2†^, Guojie Xu^1,2†^, Arit Banerjee^1,2†^, Edward S. Boyden^2,3,4,5,6,7,8^, Li-Huei Tsai^1,2,9*^

^1^Picower Institute for Learning and Memory, Massachusetts Institute of Technology, Cambridge, MA, USA

^2^Department of Brain and Cognitive Sciences, Massachusetts Institute of Technology, Cambridge, MA, USA

^3^Media Arts and Sciences, Massachusetts Institute of Technology, Cambridge, MA, USA

^4^McGovern Institute, Massachusetts Institute of Technology, Cambridge, MA, USA

^5^Department of Biological Engineering, Massachusetts Institute of Technology, Cambridge, MA, USA

^6^Koch Institute, Massachusetts Institute of Technology, Cambridge, MA, USA

^7^Center for Neurobiological Engineering, Massachusetts Institute of Technology, Cambridge, MA, USA

^8^Howard Hughes Medical Institute, Cambridge, MA, USA

^9^Broad Institute of Harvard and Massachusetts Institute of Technology, Cambridge, MA, USA

^†^These authors contributed equally to this work and share first authorship

*** Correspondence:**Li-Huei Tsai
lhtsai@mit.edu

# Supplementary Figures


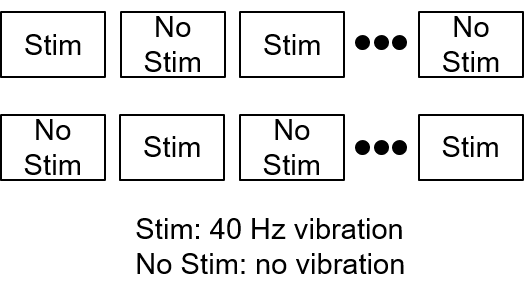


**Supplementary Figure 1**. Placement of stimulation and no stimulation speakers inside the stimulation room

**
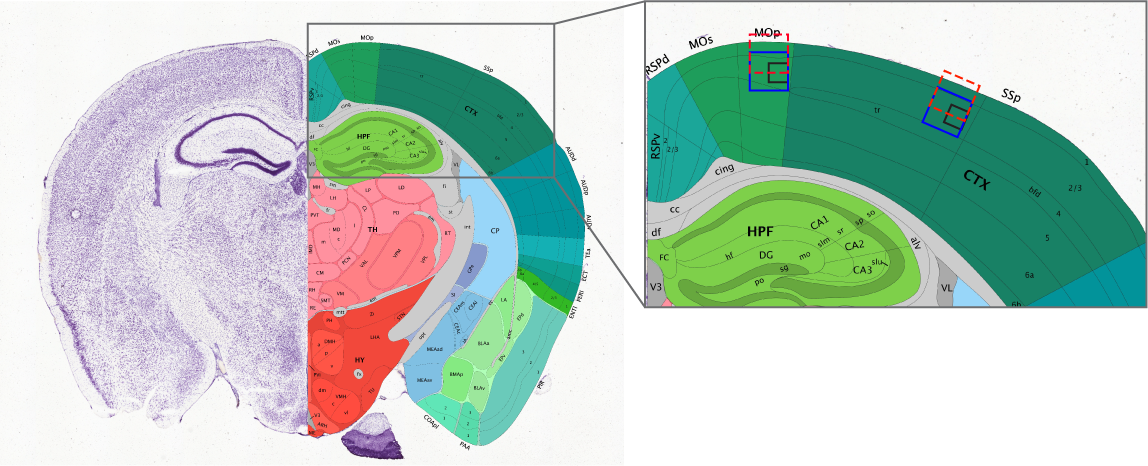
**

**Supplementary Figure 2**. Region of interest (ROI) placement for immunohistochemistry imaging

Red dashed boxes show the ROI placement for pTau (S396) imaging (Figures 2A-D), black boxes show the ROI placement for NeuN imaging (Figures 2E-G), and blue boxes show the ROI placement for c-Fos imaging (Figures 1C-E), GABBR1, vGlut1, and γH2Ax imaging (Figure 3).

**
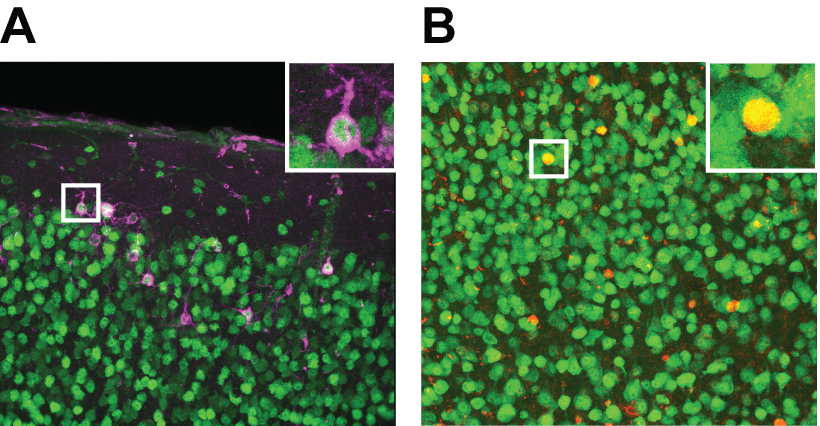
**

**Supplementary Figure 3**. Representative immunohistochemistry images showing the overlay of NeuN antibody (green) with (A) anti-pTau (S396) antibody (magenta) or (B) anti-γH2Ax antibody (red).


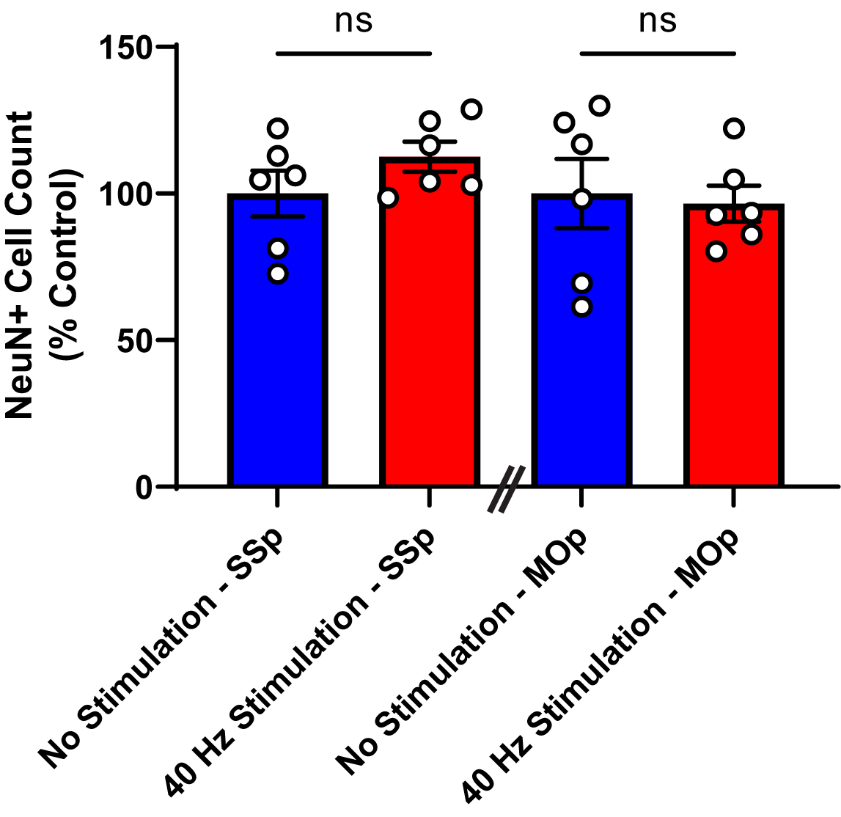


**Supplementary Figure 4.** 40 Hz vibrotactile stimulation does not significantly modify neuronal loss in SSp and MOp of CK-p25 mice

Number of NeuN-positive cells in SSp and MOp of 6-month-old CK-p25 mice after 42 days of no stimulation or 40 Hz vibrotactile stimulation, 1 hour/day, normalized to the average of no stimulation controls (n = 6 mice per group). ns = not significant; unpaired t-test. Each circle represents an individual moue, each bar represents the mean, and error bars represent SEM.
